# 

**Published:** 1983-07

**Authors:** 


### Centenary Year
### 1883–1983

### BRISTOL MEDICO-CHIRURGICAL JOURNAL

JULY 1983
VOLUME 98 (iii)
NUMBER 367

